# Current state‐of‐the‐art hyperpolarized ^13^C‐acetate‐to‐acetylcarnitine imaging is not indicative of the altered balance between glucose and fatty acid utilization associated with diabetes

**DOI:** 10.14814/phy2.12975

**Published:** 2016-09-13

**Authors:** Ulrich Koellisch, Christoffer Laustsen, Thomas S. Nørlinger, Jakob A. Østergaard, Allan Flyvbjerg, Concetta V. Gringeri, Marion I. Menzel, Rolf F. Schulte, Axel Haase, Hans Stødkilde‐Jørgensen

**Affiliations:** ^1^ Institute of Medical Engineering Technische Universit at Munchen Munich Germany; ^2^ Department of Clinical Medicine Aarhus University Aarhus Denmark; ^3^ Department of Endocrinology and Internal medicine Aarhus University Hospital Aarhus Denmark; ^4^ Danish Diabetes Academy Aarhus Denmark; ^5^ Nuklearmedizinische Klinik und Poliklinik Klinikum rechts der Isar Technische Universität München Munich Germany; ^6^ GE Global Research Munich Germany

Dear Editor,

Indeed acetate trafficking matters, however, hyperpolarized ^13^C‐acetate‐to‐acetylcarnitine is unable to detect any significant alterations between healthy controls and type‐1 diabetic rat heart, liver, and kidney, respectively in the fed state, with the current clinical setting hyperpolarized methodology.

One potential reason for this could be that hyperpolarized MR experiments are in general utilizing superphysiological substrate concentrations and are thus normally considered to perturb the normal physiological conditions. This does however not alter the main conclusion of our study, the inability to differentiate ^13^C‐acetate‐to‐acetylcarnitine conversion between diabetics and controls, thus limiting the use of the current ^13^C‐acetate methodology in diabetes patients.

It is however noteworthy that alterations in the hyperpolarized ^13^C‐acetate‐to‐acetylcarnitine conversion has been observed in skeletal muscle (10‐fold reduction) following acute hypoxia and as similarly observed in this study, a organ‐dependent difference in the ^13^C‐acetate‐to‐acetylcarnitine conversion (Jensen et al. 2009). This difference in short‐chain fatty acid metabolism is likely originating from the intraorgan difference in acetyl‐CoA synthetase isoform distribution in tissue (Jensen et al. 2009).

We agree completely with Dr. Zammit and Dr. Arduini that it is the long‐chain fatty acid acetyl transferases (carnitine palmitoyltransferases [CPT1 and CPT2]) that are maonyl‐CoA sensitive and not the carnitine acetyltransferase (generally referred to as CRAT or CAT) (Ramsay and Zammit 2004). This is an unfortunate misrepresentation adopted from the previous work (Koellisch et al. 2015). We sincerely regret this mistake, albeit a significant oversight, neither the study rationale nor the results are affected by this misrepresentation. The use of the short‐chain fatty acid acetate as an imaging biomarker has on the other hand shown success in PET, where acetate turnover is associated with oxygen consumption in both heart and kidney (Shreve et al. 1995; Juillard et al. 2007). Thus, we examined if the metabolic imbalance between the glucose utilization and fatty acid oxidation seen in diabetes would be observable in the diabetic rat in heart, liver, and kidneys by hyperpolarized ^13^C‐acetate‐to‐acetylcarnitine conversion.

As mentioned by Dr. Zammit and Dr. Arduini, the balance between fatty acid oxidation and glucose metabolism is affected by the acetyl‐CoA pool size, which is in turn regulated by the carnitine pool size, can be monitored by the acetate‐to‐acetylcarnitine conversion and is as such most likely indicative of the acetyl‐CoA synthetase activity (Bastiaansen et al. 2013).

Acetyl‐CoA synthetase substrate imbalance has been associated with hypoxia, myocardial disease, fatty acid oxidation disorders, and diabetes (Rebouche and Paulson 1986; Jensen et al. 2009).

The variability in the diabetes induction is unlikely the cause of the negative results seen in this study, as there is no correlation between the severity of diabetes and the ^13^C‐acetate‐to‐acetylcarnitine conversion. A severity‐dependent metabolic profile with hyperpolarized MR has been demonstrated with hyperpolarized [1‐^13^C]pyruvate in the same animal model (Laustsen et al. 2014a, 2013,b).

Although this study did not find acetate as a potential biomarker of the balance between glucose and fatty acid utilization with the current 3T clinical scanner and human ready hyperpolarizer, this do not rule out future improvements potentially utilizing hyperpolarized ^13^C‐acetate as an in vivo biomarker in diabetes.
