# Erratum

**DOI:** 10.14814/phy2.12999

**Published:** 2016-10-05

In Koellisch et al. (2016), the following error was published in the first paragraph.

The original letter that this response letter addresses should have been clearly referenced. The opening paragraph should have read:

“In response to the letter from Dr. Zammit and Dr. Arduini published in Physiological Reports (Zammit and Arduini 2016), we agree that indeed acetate trafficking matters, however, hyperpolarized ^13^C‐acetate‐to‐acetylcarnitine is unable to detect any significant alterations between healthy controls and type‐1 diabetic rat heart, liver, and kidney, respectively in the fed state, with the current clinical setting hyperpolarized methodology.”

This response letter (10.14814/phy2.12975) should have been published together with the letter to the Editor by Dr. Zammit and Dr. Arduini (10.14814/phy2.12997).

We apologize for the errors.
